# Chemical Reactions of Indole Alkaloids That Enable Rapid Access to New Scaffolds for Discovery

**DOI:** 10.1055/a-2048-8412

**Published:** 2023-05-11

**Authors:** Derek A. Leas, Daniel C. Schultz, Robert W. Huigens

**Affiliations:** Department of Medicinal Chemistry, Center for Natural Products Drug Discovery and Development (CNPD3), College of Pharmacy, University of Florida, 1345 Center Drive, Gainesville, FL 32610, USA

**Keywords:** indole alkaloids, yohimbine, vincamine, reserpine, chemical synthesis, ring distortion

## Abstract

This graphical review provides a concise overview of indole alkaloids and chemical reactions that have been reported to transform both these natural products and derivatives to rapidly access new molecular scaffolds. Select biologically active compounds from these synthetic efforts are reported herein.

## Graphical Abstract

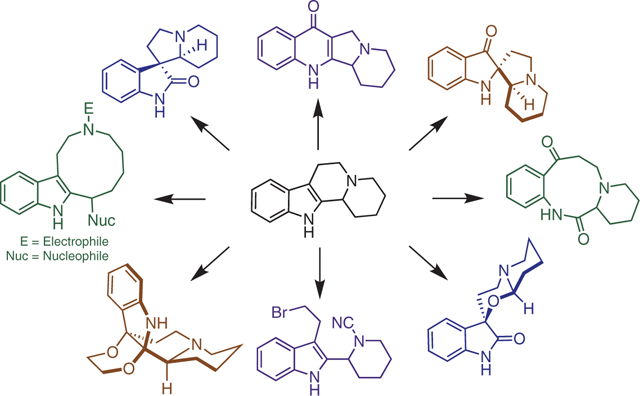

Natural products have played an essential role in medicine due to their abilities to bind to and modulate biological targets critical to disease. Vincristine, vancomycin, morphine, and paclitaxel are complex natural products with unique molecular architectures enabling exquisite drug–target interactions and therapeutic benefit to humankind. Many drug discovery programs have focused on utilizing synthetic chemistry to optimize the inherent biological activity, or pharmacology, of natural products as disease treatments; however, this graphical review focuses on synthetic transformations of indole alkaloids and relevant derivatives that would be expected to significantly alter, or re-engineer, their biological activity profiles.

Our group is developing a ring distortion platform to re-engineer the biological activities of readily available indole alkaloids using a combination of ring cleavage, ring rearrangement, and ring fusion reactions to rapidly generate diverse collections of small molecules bearing high stereochemical complexity. We hypothesize that dramatically altering the inherently complex molecular architectures of indole alkaloids will lead to new biologically active small molecules with activity profiles distinct from the parent indole alkaloid and alternative derivatives with diverse scaffolds.

Upon scanning the literature, one can find a diversity of exciting synthetic transformations that have been applied to numerous indole alkaloids and related indole-based molecules. Although these transformations have been used in total synthesis or methodology development, we view these precedented reactions as potential launching points for ring distortion chemistry. The overarching goals of this graphical review are to provide an overview of useful synthetic transformations of indole alkaloids (and related derivatives) by reaction type and for select indole alkaloids (e.g., yohimbine, vincamine).

This graphical review will begin with some basic background information related to a diversity of biologically active indole alkaloids (there are also many synthetic indole compounds of therapeutic utility in significant disease areas). Then, we will transition the graphical review to published ring cleavage and ring rearrangement transformations on indole alkaloids and derivatives. Finally, we will focus on reported transformations of select indole alkaloids (e.g., yohimbine, reserpine, catharanthine) that have been used, or could be useful, to generate novel scaffolds for drug discovery and chemical biology.

## Figures and Tables

**Figure 1:**
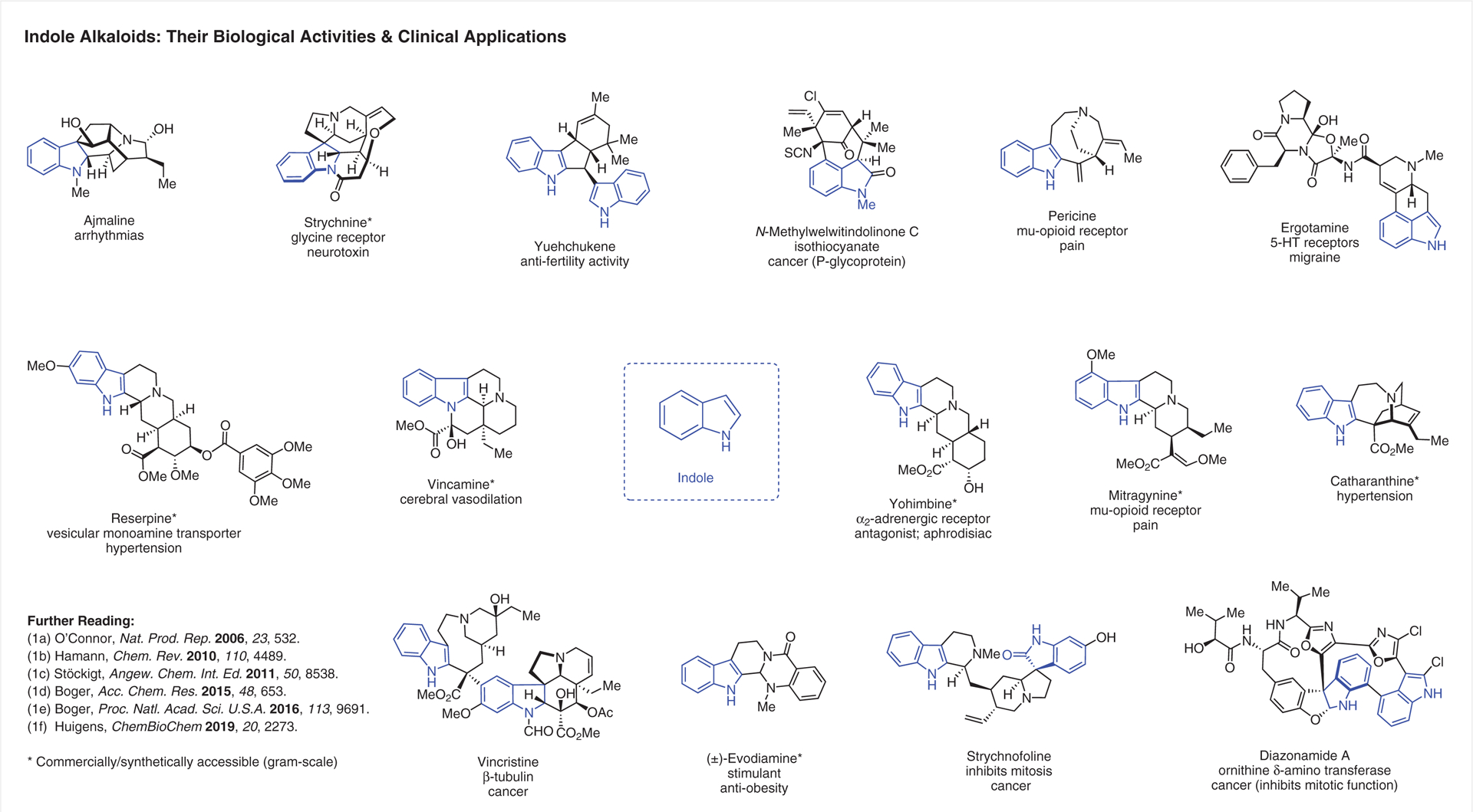
Select indole alkaloids, their biological activities and clinical applications^1a–f^

**Figure 2:**
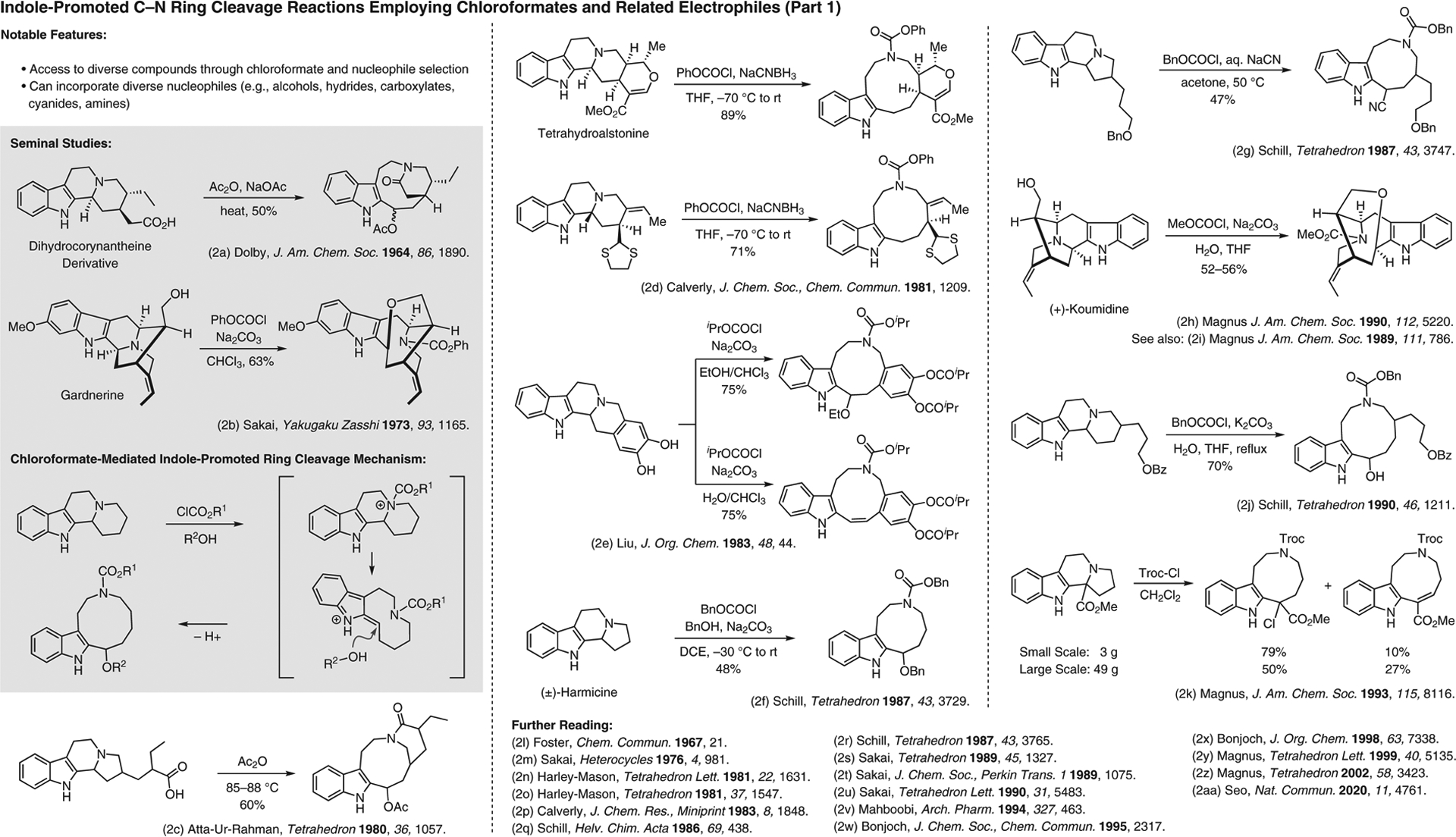
Indole-promoted C–N ring cleavage reactions employing chloroformates and related electrophiles (Part 1)^2a–2aa^

**Figure 3:**
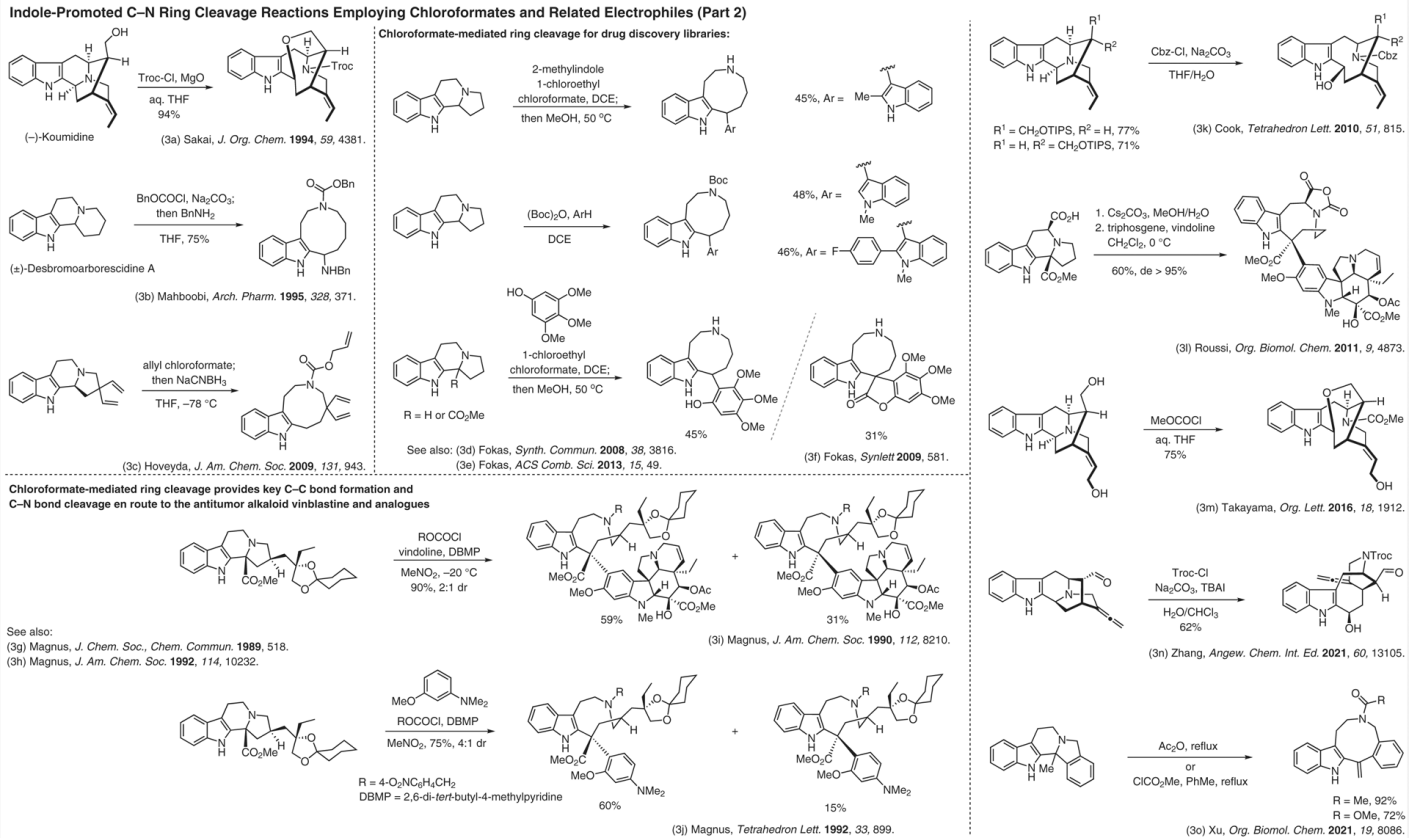
Indole-promoted C–N ring cleavage reactions employing chloroformates and related electrophiles (Part 2)^3a–o^

**Figure 4:**
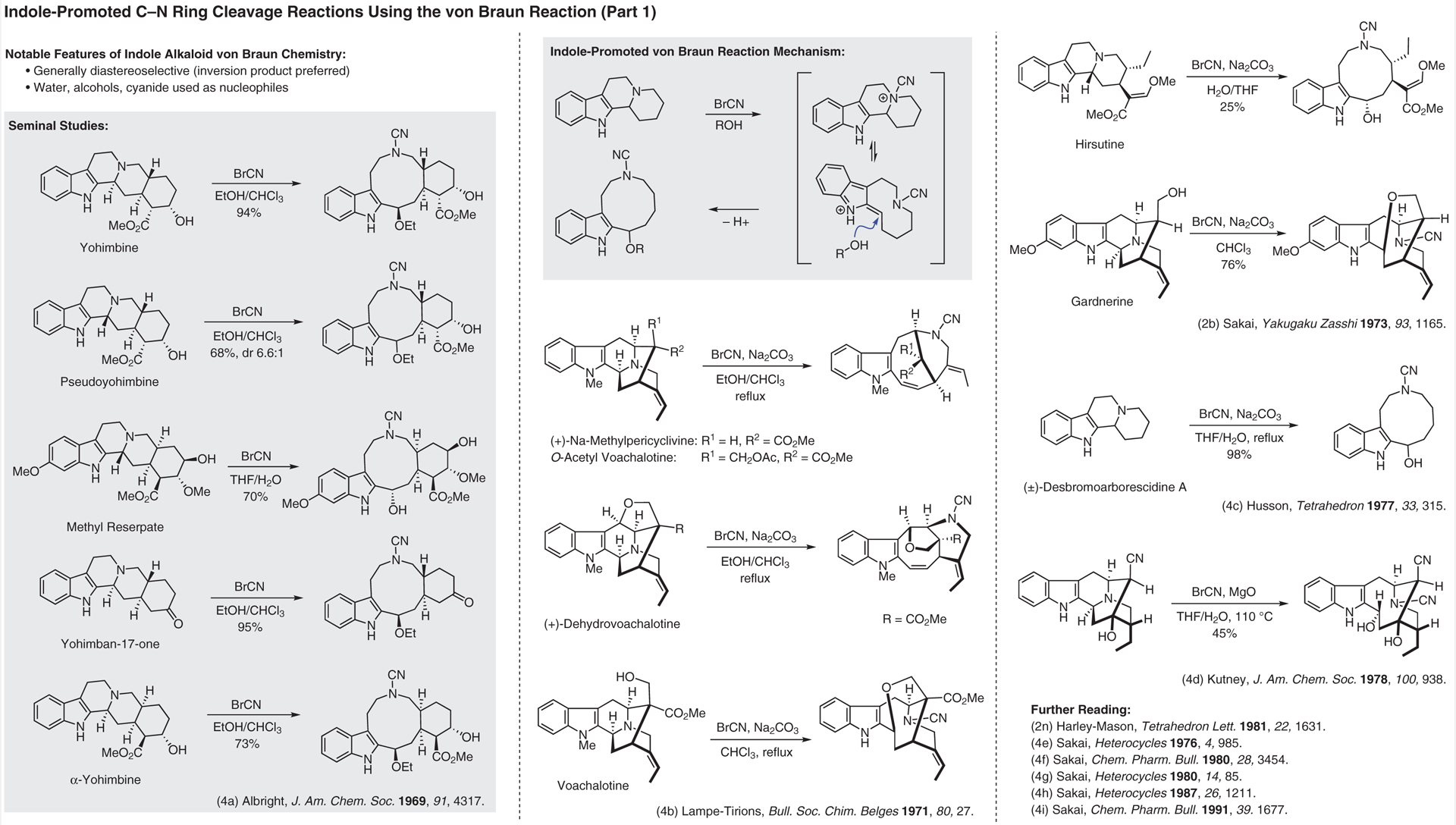
Indole-promoted C–N cleavage reactions using the von Braun reaction (Part 1)^2b,n,4a–i^

**Figure 5:**
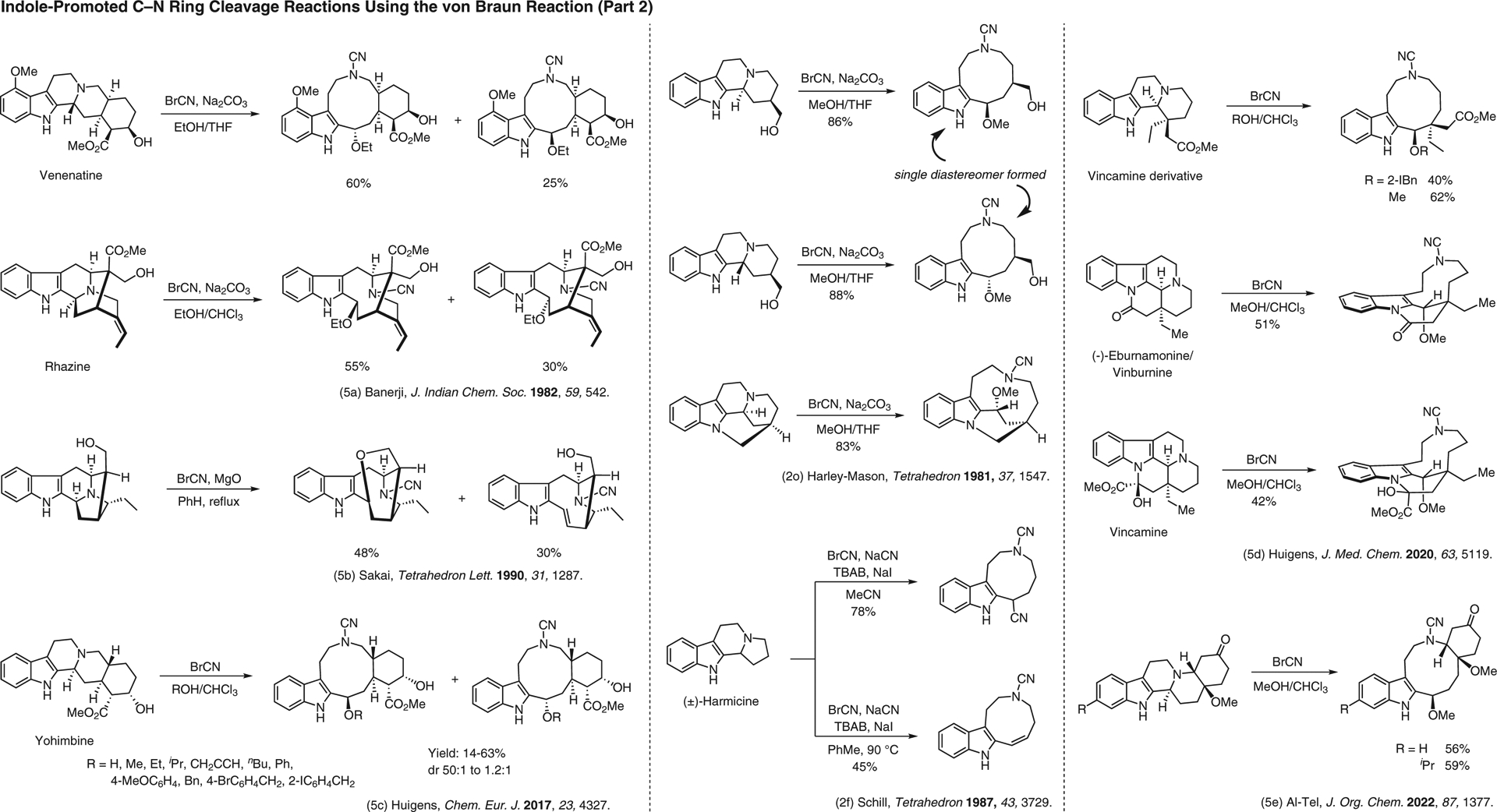
Indole-promoted C–N cleavage reactions using the von Braun reaction (Part 2)^2f,o,5a–e^

**Figure 6:**
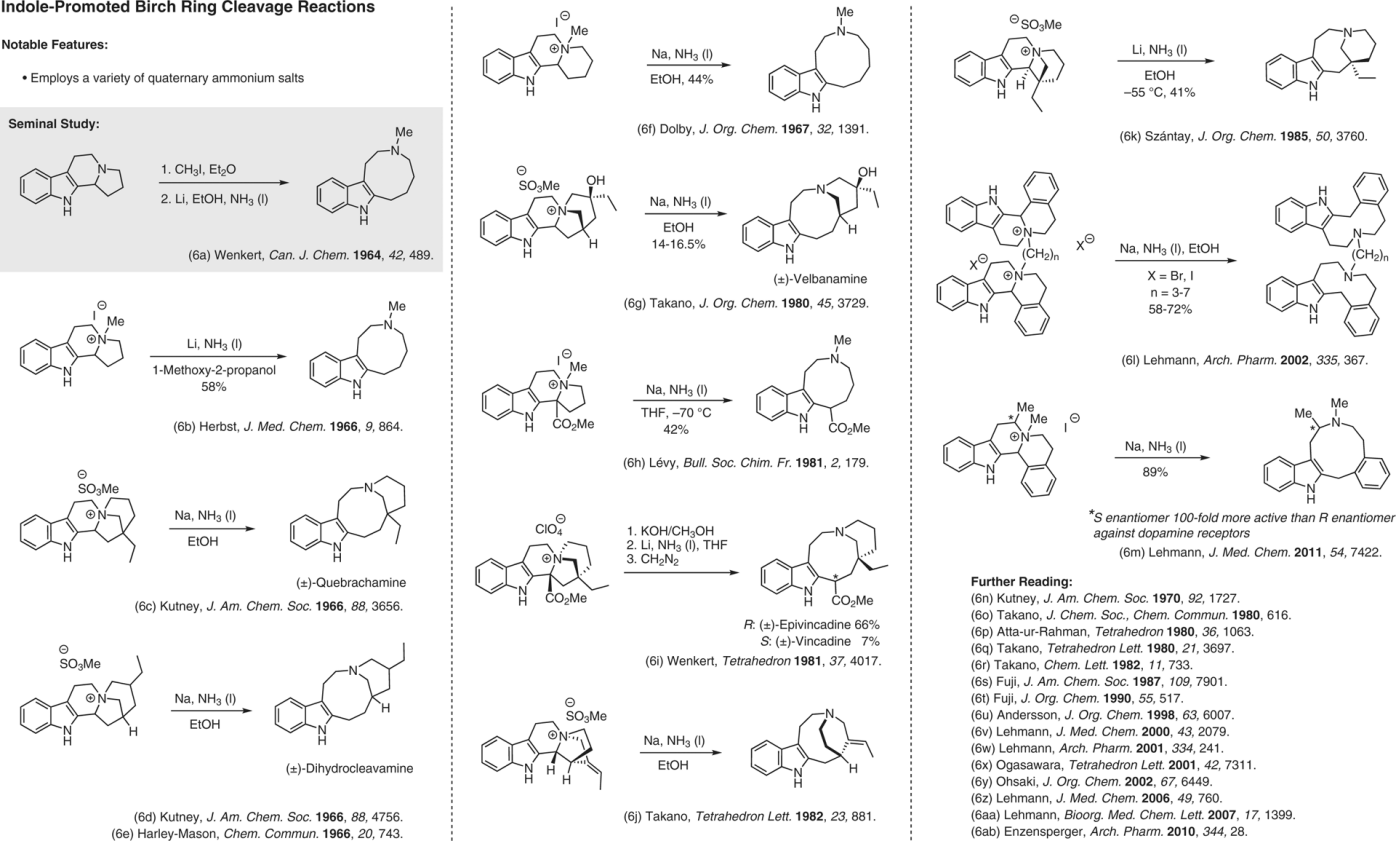
Indole-promoted Birch ring cleavage reactions^6a–ab^

**Figure 7:**
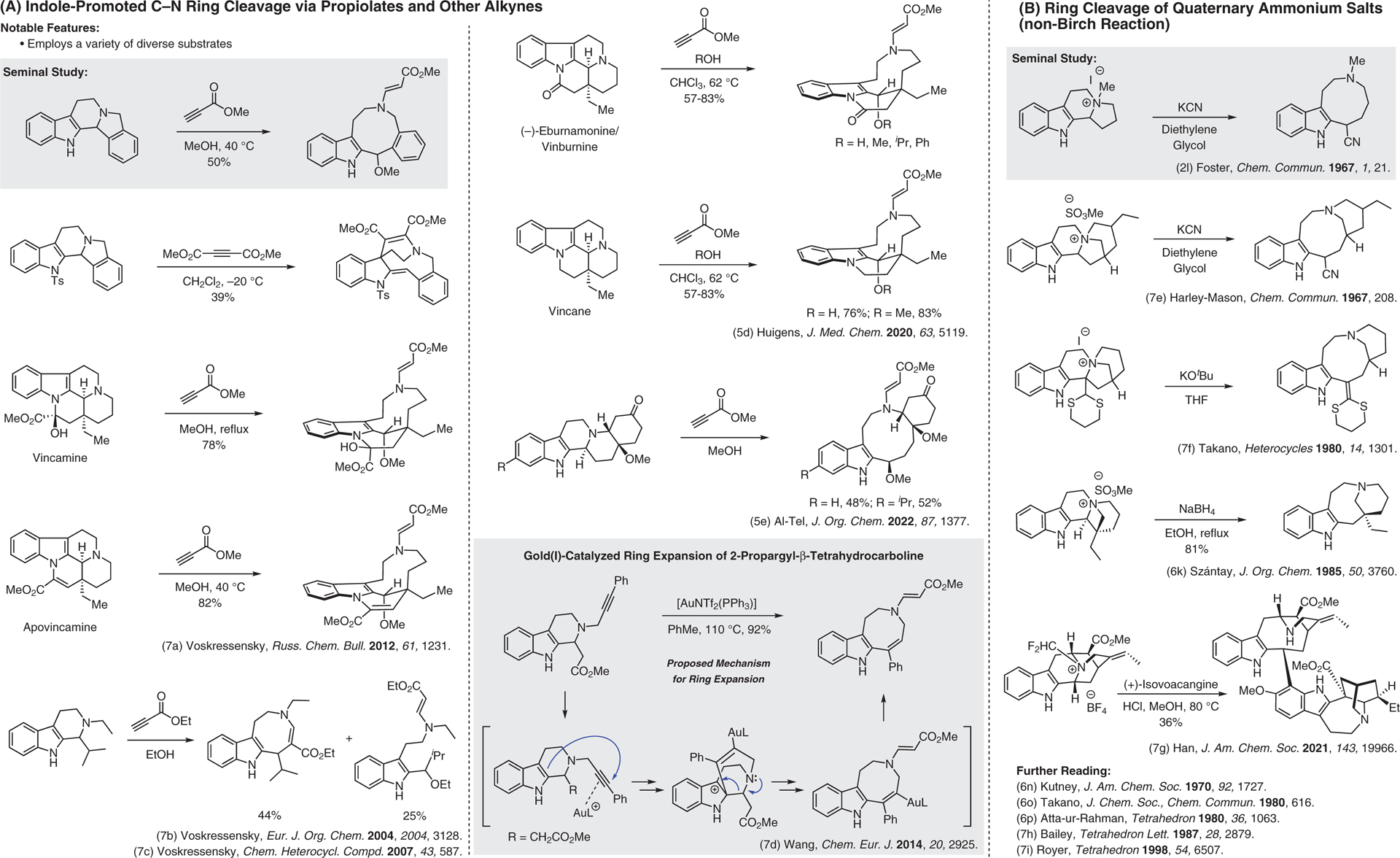
(A) Indole-promoted C–N ring cleavage via propiolates and other alkynes. (B) Ring cleavage of quaternary ammonium salts (non-Birch reaction).^2l,5d,e,6o–p,7a–i^

**Figure 8:**
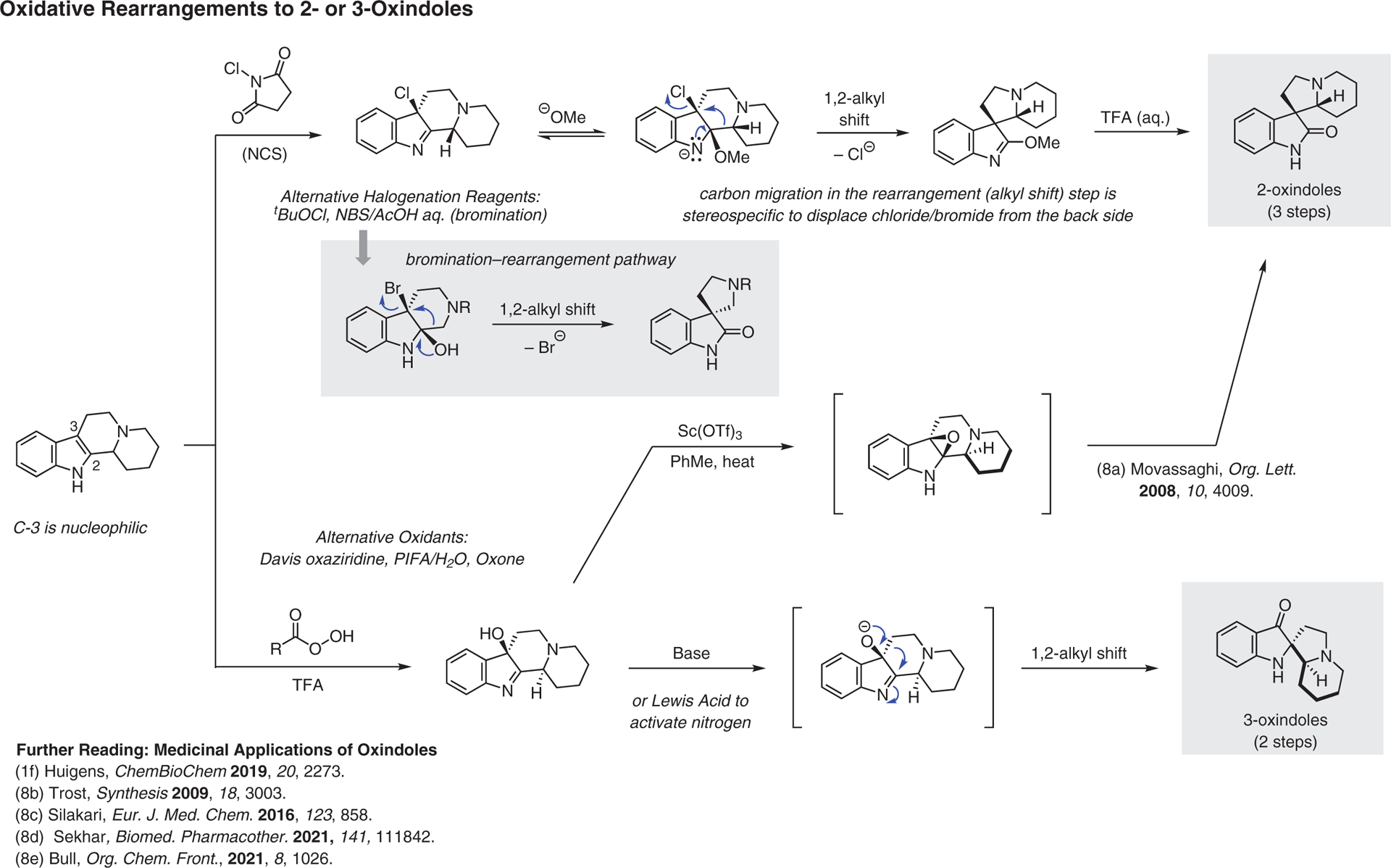
Oxidative rearrangements to 2- or 3-oxindoles^1f,8a–e^

**Figure 9:**
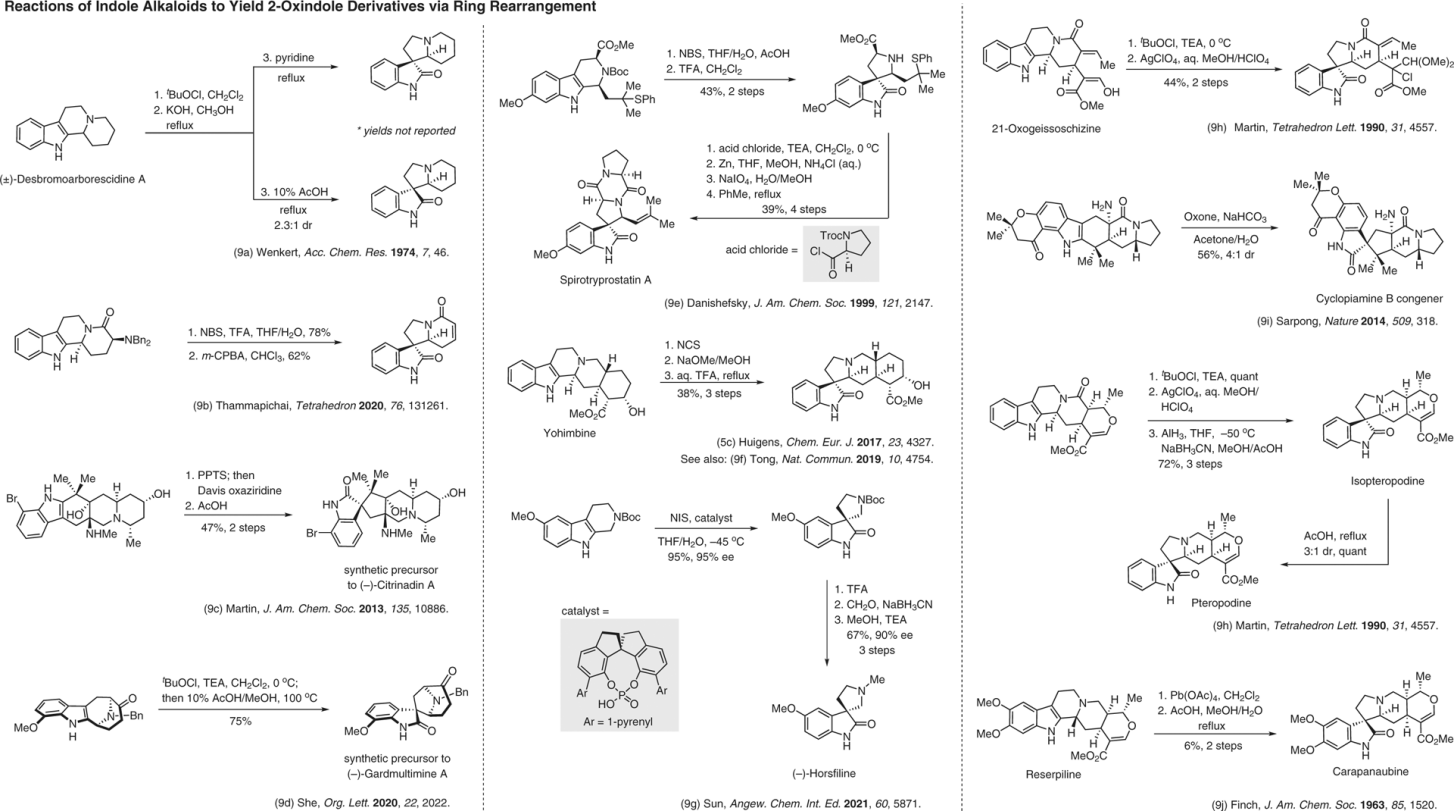
Reactions of indole alkaloids to yield 2-oxindole derivatives via ring rearrangement^5c,9a–j^

**Figure 10:**
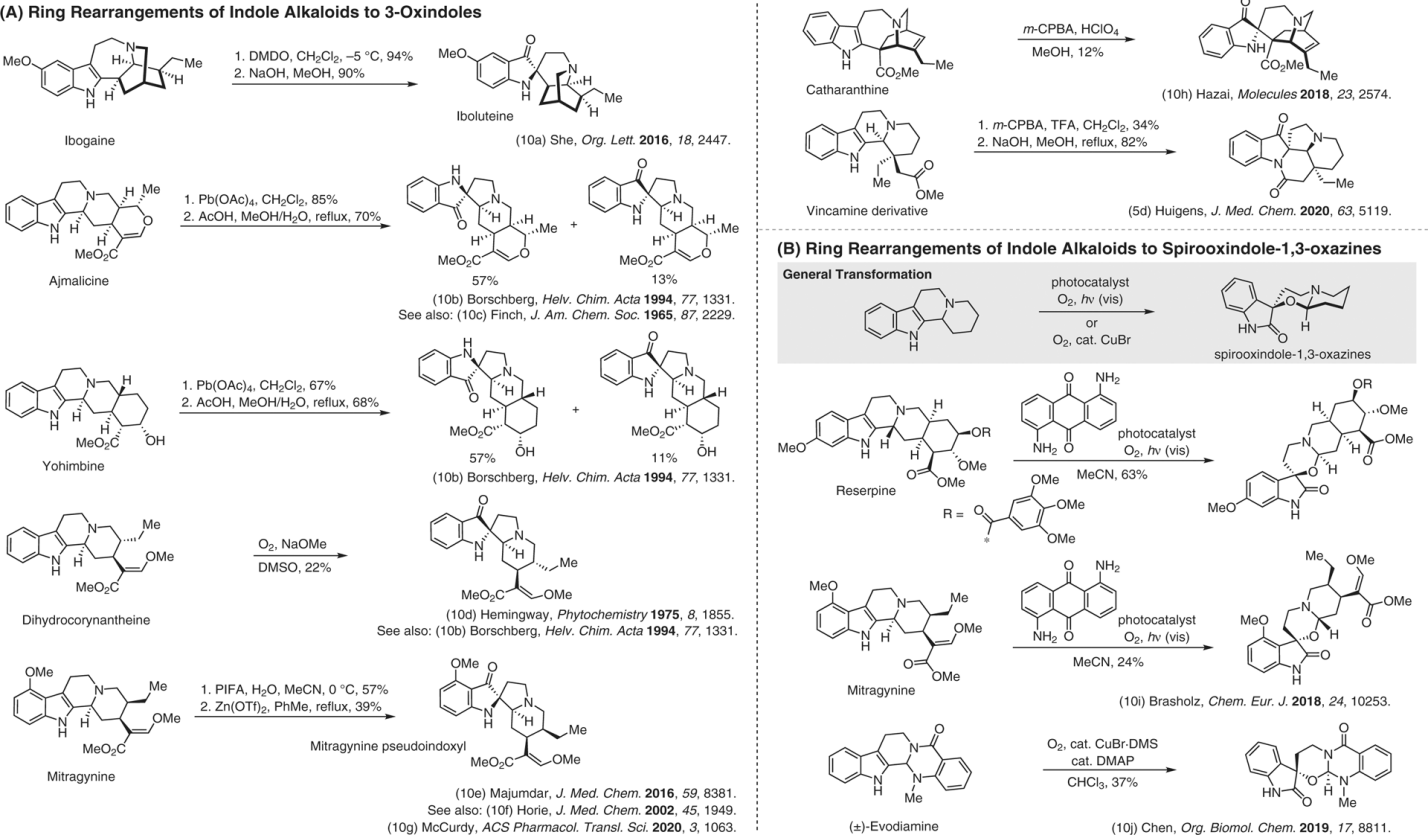
(A) Ring rearrangements of indole alkaloids to 3-oxindoles. (B) Ring rearrangements of indole alkaloids to spirooxindole-1,3-oxazines.^5d,10a–j^

**Figure 11:**
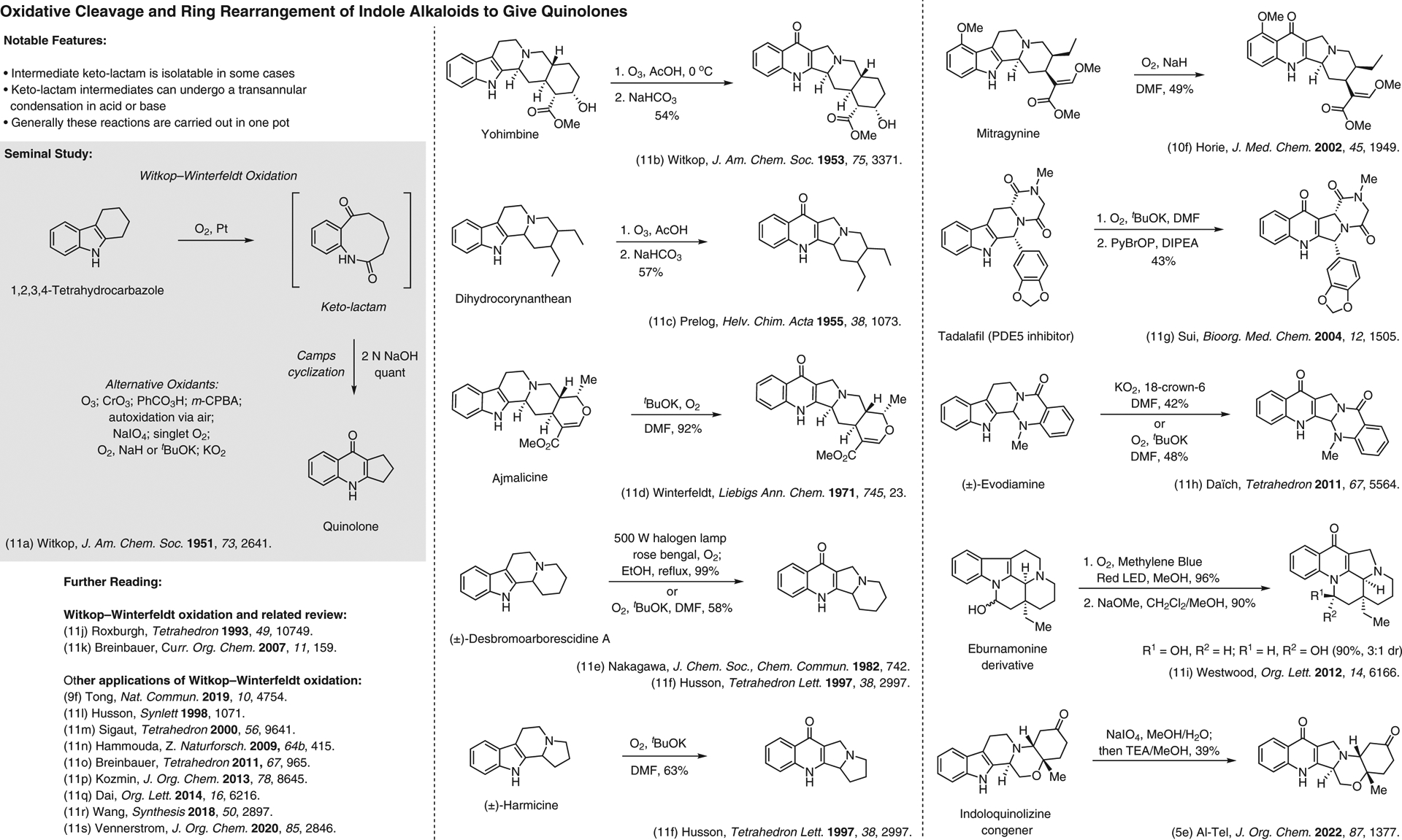
Oxidative cleavage and ring rearrangement of indole alkaloids to give quinolones^5e,9f,10f,11a–s^

**Figure 12:**
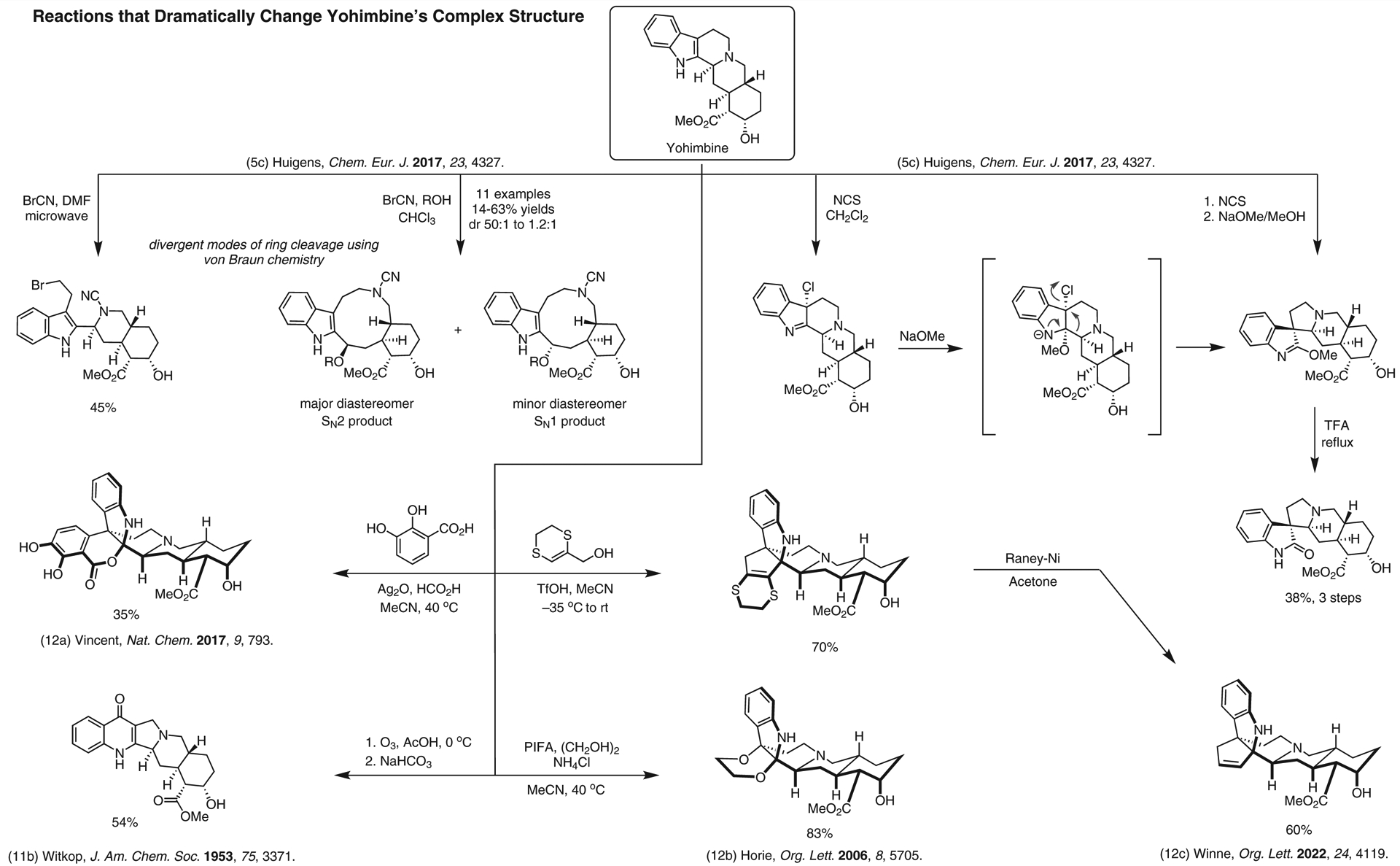
Reactions that dramatically change yohimbine’s complex structure^5c,11b,12a–c^

**Figure 13:**
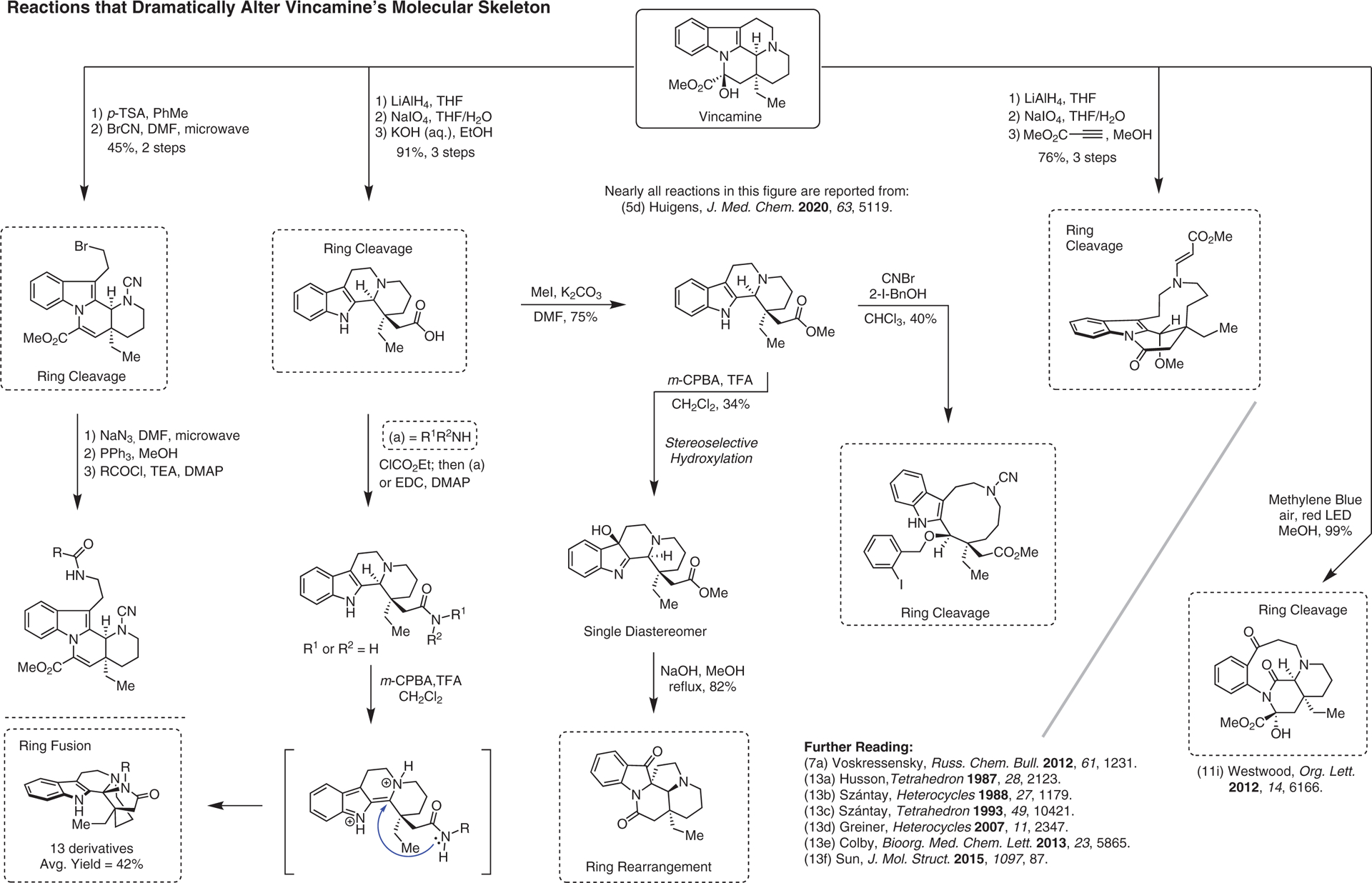
Reactions that dramatically alter vincamine’s molecular skeleton^5d,7a,11i,13a–f^

**Figure 14:**
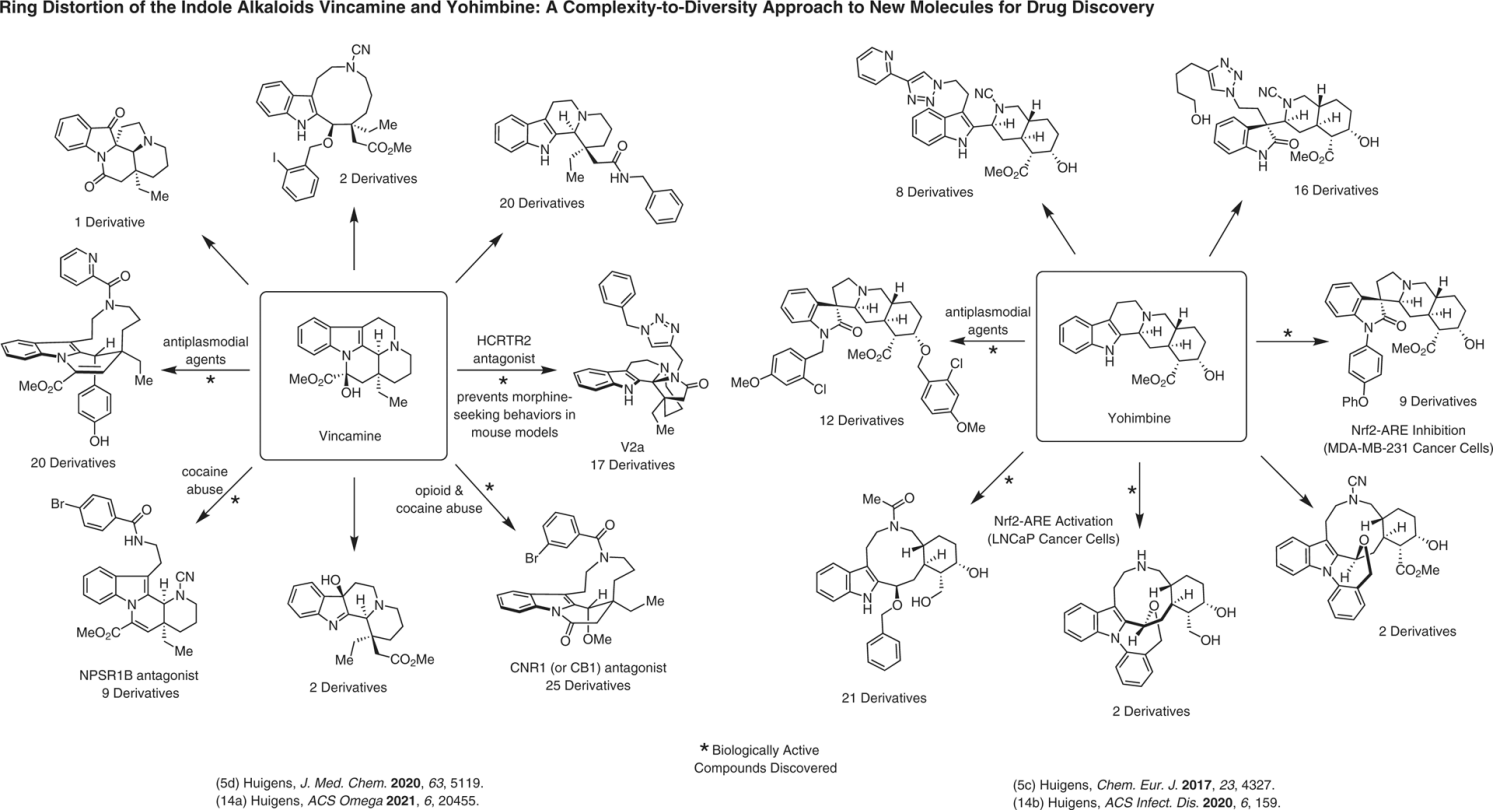
Ring distortion efforts of vincamine and yohimbine, and the discovery of biologically active small molecules in significant disease areas (e.g., cancer, opioid addiction, malaria)^5c,d,14a,b^

**Figure 15:**
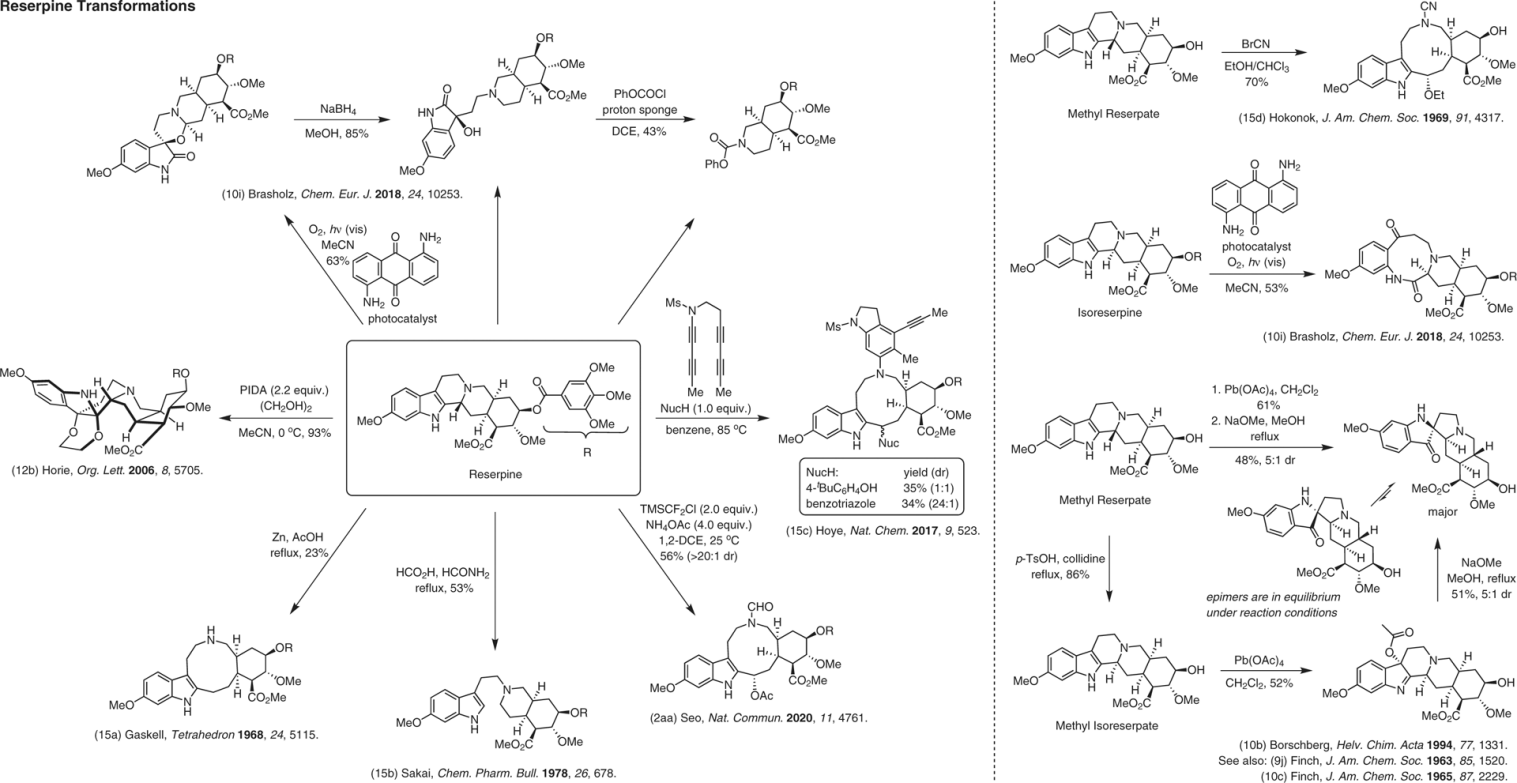
Chemical reactions of reserpine that significantly change its architecture^2aa,9j,10b,c,i,12b,15a–d^

**Figure 16:**
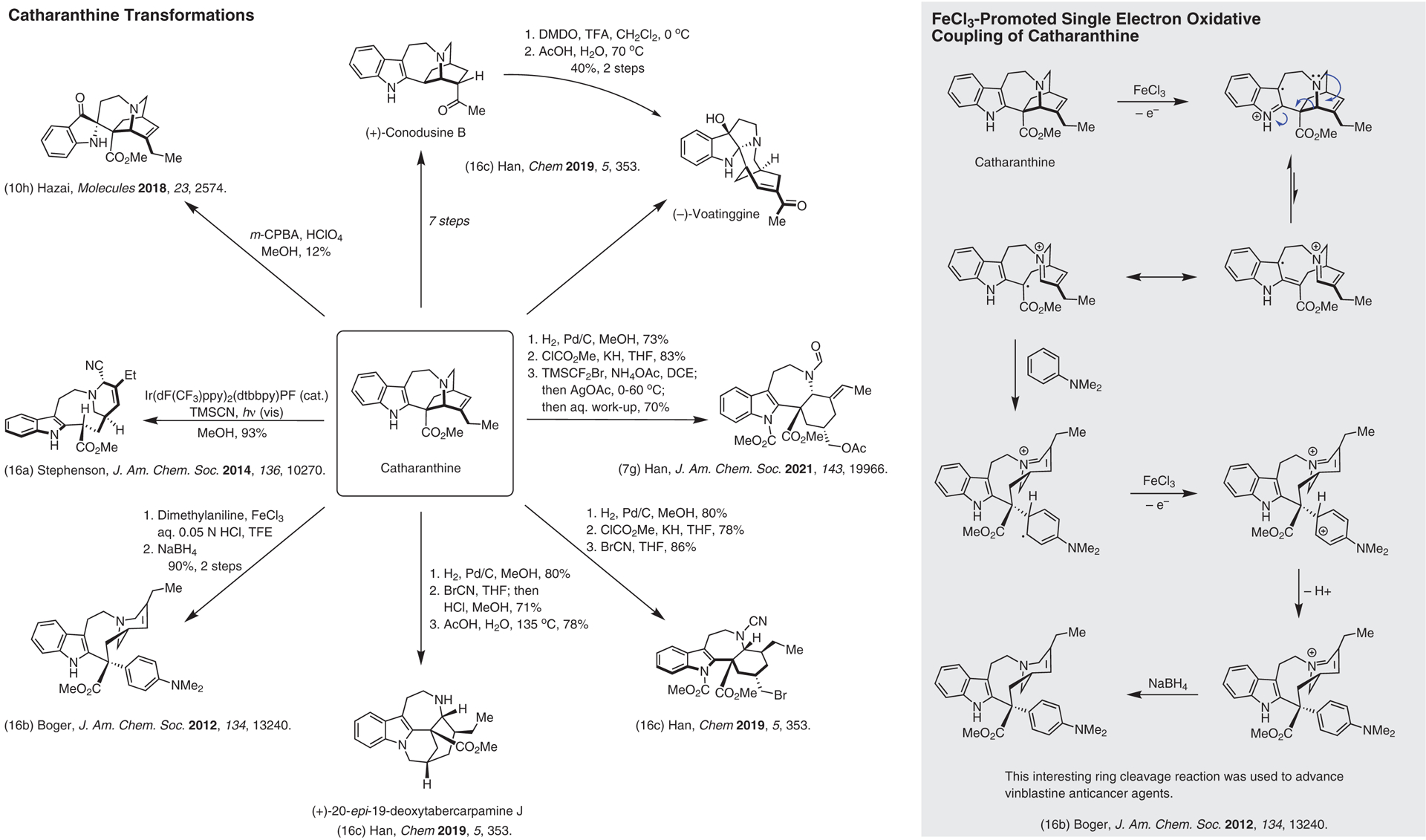
Reactions that alter catharanthine’s complex structure^7g,10h,16a–c^

**Figure 17:**
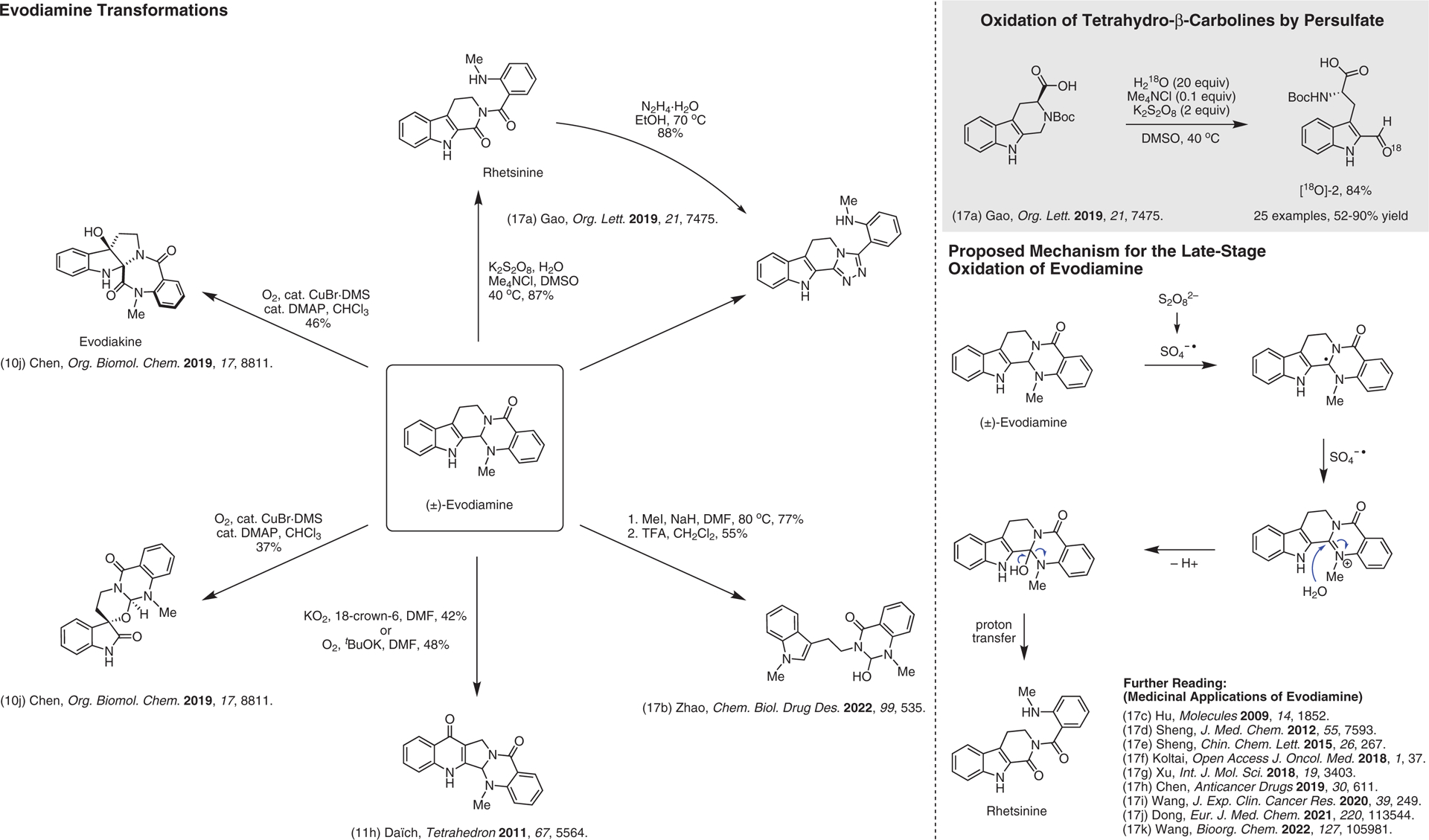
Chemical reactions reported to dramatically change evodiamine’s scaffold^10j,11h,17a–k^
